# Application of circulating tumour DNA in terms of prognosis prediction in Chinese follicular lymphoma patients

**DOI:** 10.3389/fgene.2023.1066808

**Published:** 2023-04-20

**Authors:** Mengjing Zhao, Qingjuan Li, Jing Yang, Min Zhang, Xiaolan Liu, Hongwei Zhang, Yunpeng Huang, Jing Li, Jiangping Bao, Jingfang Wang, Jun Du, Tao Guan, Liping Su

**Affiliations:** ^1^ Department of Biochemistry and Molecular Biology, School of Basic Medicine, Shanxi Medical University, Taiyuan, China; ^2^ Department of Hematology, Shanxi Province Cancer Hospital, Shanxi Hospital Affiliated to Cancer Hospital, Chinese Academy of Medical Sciences, Cancer Hospital Affiliated to Shanxi Medical University, Taiyuan, China; ^3^ Department of Pathology, Shanxi Province Cancer Hospital, Shanxi Hospital Affiliated to Cancer Hospital, Chinese Academy of Medical Sciences, Cancer Hospital Affiliated to Shanxi Medical University, Taiyuan, China; ^4^ Department of Microbial and Biochemical Pharmacy, School of Pharmaceutical Sciences, Sun Yat-sen University, Guangzhou, China

**Keywords:** follicular lymphoma, circulating tumour DNA, targeted next-generation sequencing, mutation, prognosis

## Abstract

**Background:** Follicular lymphoma (FL), an indolent non-Hodgkin lymphoma (NHL), is generally incurable. Favourable prognosis and durable remission are crucial for FL patients. The genetic mutation spectrum provides novel biomarkers for determining the prognosis of FL patients, but its detection is easily affected by the collection of tumour tissue biopsies. In this study, we aimed to describe the mutational landscape of FL using circulating tumour DNA (ctDNA) samples and to explore the relationship between mutations and prognostic indicators of clinical outcome in patients with newly diagnosed follicular lymphoma and the prognostic value of such mutations.

**Methods:** A total of 28 patients with newly diagnosed FL were included in this study. A targeted NGS-based 59-gene panel was used to assess the ctDNA mutation profiles. Differences in clinical factors between patients carrying mutations and those without mutations were analysed. We also explored the relationship between gene mutation status, mean VAFs (variant allele frequencies) and clinical factors. The Kaplan‒Meier method was applied to analyse the overall survival (OS) and progression-free survival (PFS) of patients carrying mutations and those without mutations.

**Results:** ctDNA mutations were detectable in 21 (75%) patients. The most commonly mutated genes were CREBBP (54%, 15/28), KMT2D (50%, 14/28), STAT6 (29%, 8/28), CARD11 (18%, 5/28), PCLO (14%, 4/28), EP300 (14%, 4/28), BCL2 (11%, 3/28), and TNFAIP3 (11%, 3/28), with a mutation frequency of >10%. Patients with detectable ctDNA mutation tended to present with advanced Ann Arbor stage (III-IV) (*p* = 0.009), high FLIPI risk (3–5) (*p* = 0.023) and severe lymph node involvement (No. of involved areas ≥5) (*p* = 0.02). In addition, we found that the mean VAF was significantly higher in patients with advanced Ann Arbor stage, high-risk FLIPI, elevated lactate dehydrogenase (LDH: 0–248U/L), advanced pathology grade, bone marrow involvement (BMI) and lymph node involvement. Additionally, KMT2D, EP300, and STAT6 mutations were associated with inferior PFS (*p* < 0.05).

**Conclusion:** We described the ctDNA mutation landscapes in Chinese patients with newly diagnosed FL and found that ctDNA VAF means reflect tumour burden. Moreover, PFS was shorter in patients with KMT2D, EP300 and STAT6 mutations.

## 1 Introduction

Follicular lymphoma (FL) is the second most common subtype of non-Hodgkin lymphoma (NHL), accounting for approximately 20% of all lymphomas in the United States and Western Europe (Teras et al., 2016; Freedman and Jacobsen, 2020). FL is a heterogeneous disease with variable outcomes that is associated with different genomic alterations (Pasqualucci et al., 2011). It has been concluded that FL is regulated by genes related to histone modifications, cell-signalling pathways, transcription factors and chromatin remodelling (Morin et al., 2011; Shih et al., 2012; Green, 2018). Understanding the genomic landscape of FL will be helpful for diagnosis, prognosis prediction and the development of precision therapy. Currently, tissue biopsy is the gold standard for pathological diagnosis and identification of genetic variants, but invasive biopsies have significant limitations. Tissue biopsies carry procedural risks and cannot account for spatial inter-and intratumor heterogeneity due to sampling from only one location in a single tumour lesion (Wang et al., 2016). Therefore, a non-invasive and reliable method is needed to better characterize genetic alterations.

Analysis of circulating tumour DNA (ctDNA) is the leading liquid biopsy approach in lymphomas and can potentially improve on the limitations of tissue biopsy (Costa & Schmitt, 2019). ctDNA is the DNA fragment derived from tumour cells, and it accounts for approximately 0.01% of cfDNA (cell-free DNA) (Schwarzenbach et al., 2011). Analysing somatic mutations in cell-free DNA (cfDNA) to identify ctDNA can provide the genetic profiles of tumour tissues. Hohaus S et al. first demonstrated that ctDNA is a quantified tumour-specific biomarker in NHLs and Hodgkin’s lymphoma (HL) by real-time PCR(Hohaus et al., 2009). It has been reported that ctDNA could be a novel and sensitive cancer biomarker that can be used for the diagnosis, follow-up of treatment and prognosis of various cancers (Melani and Roschewski, 2016; Roschewski et al., 2016; Cescon et al., 2020). Evidence has demonstrated that ctDNA can reflect tumour biopsies in regards to somatic mutations in diffuse large B cell lymphoma (DLBCL) because of the high concordance of mutation profiles between ctDNA samples and tissue samples, and the use of ctDNA can overcome the issue of intratumor spatial heterogeneity (Eskandari et al., 2019; Liu et al., 2020). However, there are few studies on the application of ctDNA in FL. Therefore, it is necessary to confirm the characteristics of the ctDNA mutation spectrum in Chinese FL patients and its clinical value. Moreover, along with the rapid advances in next-generation sequencing (NGS) technology, ctDNA provides new opportunities for a better understanding of the molecular mechanisms of FL.

In this study, targeted NGS was applied to draw a ctDNA mutational landscape in newly diagnosed FL patients. Then, we assessed the relationship between ctDNA mutations and clinical pathological features and the roles of ctDNA mutations, including mutation status and mean VAF, in the overall survival (OS) and progression-free survival (PFS) of these patients.

## 2 Materials and methods

### 2.1 Patients

A total of 28 newly diagnosed FL patients were enrolled at Shanxi Cancer Hospital from December 2018 to June 2021. Patients were considered eligible if they were aged >18 years and had histologically confirmed FL according to the 2016 WHO Classification of Tumors of Hematopoietic and Lymphoid Tissue (Swerdlow et al., 2016). None of the patients had family history. The disease was staged based on the 2014 Lugano classification, and the FL international prognostic index (FLIPI) was applied for risk stratification. Bone marrow involvement was assessed by flow cytometry combined with IG rearrangement and PET-CT. All patients were treated with the R-CHOP regimen (rituximab, cyclophosphamide, doxorubicin, vincristine, and prednisone). We retrospectively analysed the clinical data of these patients and followed them up to March 2022. The treatment response, including CR (complete response), PR (partial response), SD (stable disease) and PD (progressive disease), was assessed by CT/MRI and PET/CT according to local guidelines after two to four treatment cycles.

All study activities were approved by the Ethics Committee of Shanxi Cancer Hospital (Ethical approval No.202236), and informed consent was obtained in accordance with the Declaration of Helsinki.

### 2.2 DNA extraction and targeted sequencing

Peripheral blood samples were collected before treatment using EDTA-containing tubes and centrifuged immediately at a speed of 820 g for 10 min to separate plasma, and then the plasma samples were centrifuged at 20,000 × *g* for 10 min. Next, cfDNA was extracted using a QIAamp Circulating Nucleic Acid Kit (QIAGEN, Germany) according to the manufacturer’s instructions. Subsequently, the mutation profiles of each cfDNA sample were detected using the targeted NGS-based 59-gene panel (Shanghai Rightongene Biotech Co., Ltd., Shanghai, China) with an Illumina NovaSeq 5,000 (2 × 150-paired-end reads) and the target regions for each gene assay are coding exons and splice sites. This method is based on our previous published research on DLBCL (Guan et al., 2022). Mutations were filtered out if the VAF ranged from 45% to 55% or 95%, as such VAFs were considered to indicate germline mutations. The VAF was defined as the number of mutant molecules at a specific nucleotide location over the total number of molecules present in the background at a specific given genomic location. The mean VAF was calculated as follows: Mean VAF = The sum of the VAF of all mutations/The number of mutations.

### 2.3 Statistical analysis

Categorical data were compared with Fisher’s exact or chi-squared test, and continuous data were compared using a two-tailed paired Mann‒Whitney *U* test. Progression-free survival (PFS) was defined as the period from the date of treatment onset to the date of progression, last follow-up, or death of any cause. Overall survival (OS) was calculated from the date of treatment onset to the date of death of any cause or last follow-up. Survival analysis was performed by the Kaplan‒Meier method, and survival differences between groups were analysed using the log-rank test. All statistical analyses were conducted with IBM SPSS software (26.0), and GraphPad Prism (9.0) was used for drawing. Probability values < 0.05 were considered significant.

## 3 Results

### 3.1 ctDNA mutation profiles of patients with newly diagnosed FL

**FIGURE 1:**
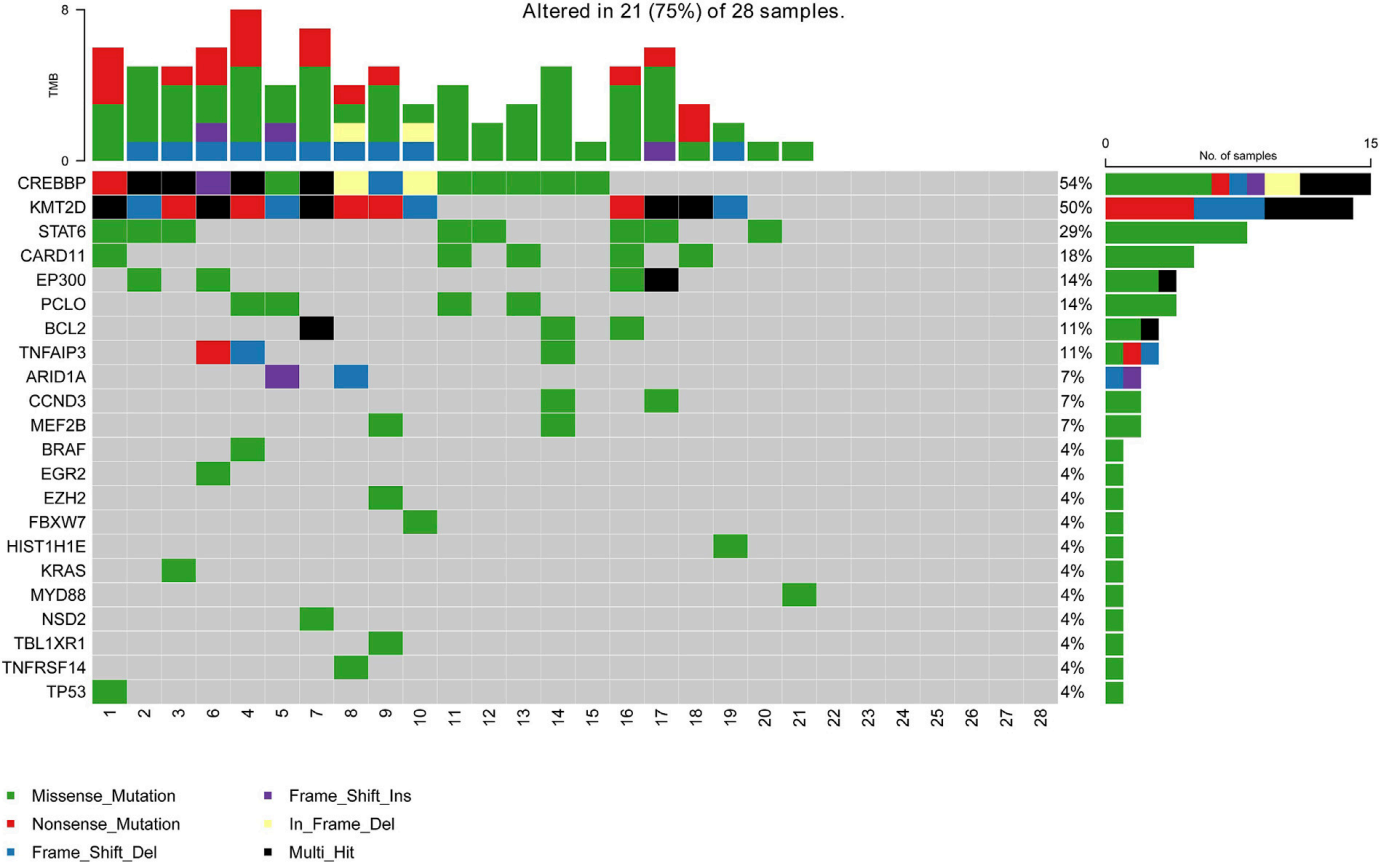
Mutation landscape of the ctDNA samples in newly diagnosed FL patients. The *X*-axis is the sample of individual patients. The upper histogram represents the number of mutated genes per sample. The *Y*-axis on the left shows the mutated genes, and the percentages on the right chart represent the mutation frequency of mutated genes.

A total of 28 newly diagnosed FL patients with valid targeted NGS data were included in this study, of which 21 patients (75%) carried ctDNA mutations. Eighty-six somatic mutations were detected, and the median mutation per sample was 4 (range from 1 to 8). The most frequently mutated genes in the whole cohort were CREBBP (54%, 15/28), KMT2D (50%, 14/28), STAT6 (29%, 8/28), CARD11 (18%, 5/28), PCLO (14%, 4/28), EP300 (14%, 4/28), BCL2 (11%, 3/28) and TNFAIP3 (11%, 3/28), with a mutation frequency of >10% (Figure 1). Additionally, pathway enrichment analysis for these mutations was performed, which showed that BCR/NF-κB signalling, epigenetic regulators and JAK/STAT signalling were universally activated in FL (Supplementary Figure S1).

### 3.2 Relationship between clinicopathologic features and mutation status

Here, we analysed the relationship between prognostic indicators and ctDNA mutation status in newly diagnosed FL patients. Patients with detectable ctDNA mutations were significantly enriched in III-IV Ann Arbor stage (85.7% vs 28.6%, *p* = 0.009) and high-risk FLIPI (3–5) (52.45% vs 0, *p* = 0.023) and were inclined to have ≥5 lymph node-involved areas (81.0% vs 28.6%, *p* = 0.020) compared to patients without detected mutations in the plasma samples (Table 1). These results indicated that the ctDNA mutation status of newly diagnosed FL patients was closely associated with patient stage, FLIPI and number of lymph node-involved areas. It is worth noting that patients with ctDNA mutations were significantly enriched in lower pathological grades (grades 1–2) (81.0% vs 28.6%, *p* = 0.020).

**TABLE 1:** The clinicopathologic features of FL patients with mutation or without mutation.

Clinical parameters	Status of ctDNA (n = 28)		χ^2^	*p*-Value
	Mut, n (%)	Wt, n (%)		
**Gender**				
Male	13 (61.9)	5 (71.4)	0.000	1.000
Female	8 (38.1)	2 (28.6)		
**Age**				
<60	15 (71.4)	5 (71.4)	0.000	1.000
≥60	6 (28.6)	2 (28.6)		
**Ann Arbor stage**				
I-II	3 (14.3)	5 (71.4)	5.833	**0.009**
III-IV	18 (85.7)	2 (28.6)		
**Pathology Classification**				
1–2 grade	17 (81.0)	2 (28.6)	4.421	**0.020**
3 grade	4 (19.0)	5 (71.4)		
**FLIPI-1 score**				
0–2	10 (47.6)	7 (100)	4.403	**0.023**
3–5	11 (52.4)	0 0)		
**LDH (0–248 U/L)**				
Elevated	7 (33.3)	0 0)	1.587	0.141
Normal	14 (66.7)	7 (100)		
**β2-M (1–2.3 mg/L)**				
Elevated	12 (57.1)	4 (57.1)	0.000	1.000
Normal	9 (42.9)	3 (42.9)		
**B symptoms**				
Positive	8 (38.1)	2 (28.6)	0.000	1.000
Negative	13 (61.9)	5 (71.4)		
**Splenomegaly**				
Positive	10 (47.6)	1 (14.3)	1.248	0.191
Negative	11 (52.4)	6 (85.7)		
**No. of involved areas ≥5**				
Positive	17 (81.0)	2 (28.6)	4.421	**0.020**
Negative	4 (19.0)	5 (71.4)		
**Bone marrow involvement**				
Positive	11 (52.4)	1 (14.3)	1.750	0.184
Negative	10 (47.6)	6 (85.7)		
**Interim assessment**				
CR	13 (61.9)	5 (71.4)	0.000	1.000
no-CR	8 (38.1)	2 (28.6)		

### 3.3 Correlation between clinical indicators and the ctDNA mean VAF

**FIGURE 2:**
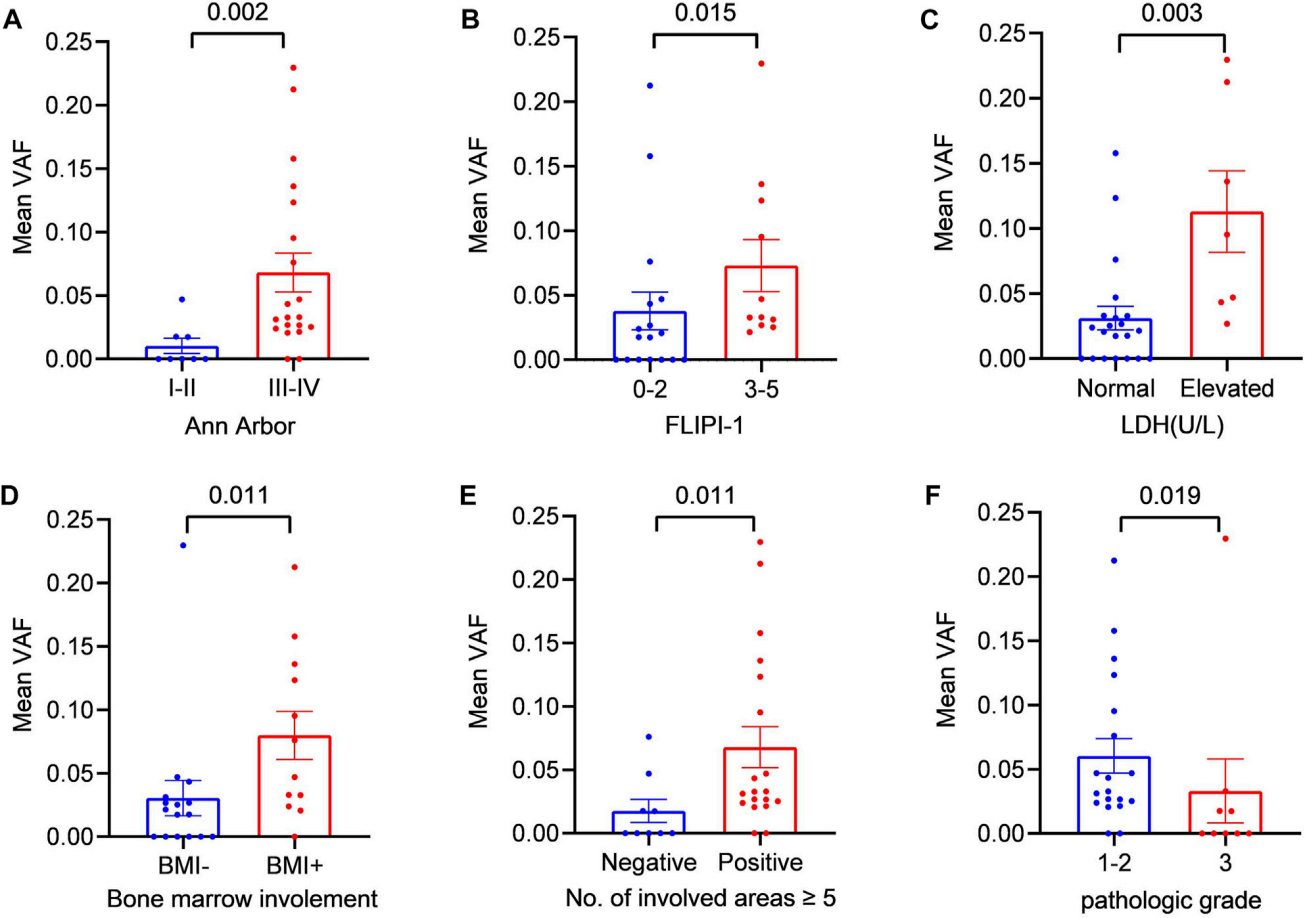
Relationship between the mean VAF and the clinical features of patients with newly diagnosed FL **(A)** Patients were divided into two groups according to Ann Arbor staging: the early-stage (I + II) group and the late-stage (III + IV) group **(B)** Statistical comparison between the low-FLIPI (0–2) group and the high-FLIPI (3–5) group was performed **(C)** Patients were divided into two groups according to LDH level: the elevated LDH group and the normal LDH group (ULN) **(D)** Patients were divided into two groups according to whether bone marrow was involved: BMI (+) and BMI (−) **(E)** Statistical comparison between the group with ≥5 lymph node-involved areas and the group with <5 lymph node-involved areas was performed. **(F)** Statistical comparison between the grade 1–2 cases and grade 3 cases was performed.

To further explore the clinical applications and value of ctDNA in newly diagnosed FL patients, we analysed the relationship between clinical indicators associated with prognosis and ctDNA levels. Here, we used mean VAFs (variant allele frequencies) to refer to mutation levels in ctDNA. As shown in Table 2, a comprehensive review and data analysis revealed that the VAF was closely correlated with poor clinical prognostic indicators, as a higher ctDNA mean VAF was associated with more advanced stage (*p* = 0.002; Figure 2A), high-risk FLIPI-1 (*p* = 0.015, Figure 2B) and elevated serum LDH levels (*p* = 0.003, Figure 2C). In addition, the ctDNA mean VAF was also significantly higher in patients with BMI (*p* = 0.011, Figure 2D) and severe lymph nodal involvement (*p* = 0.011, Figure 2E). However, the ctDNA mean VAF was significantly lower in grade 3 patients than in grade 1–2 patients (*p* = 0.019, Figure 2F). These results effectively suggest that ctDNA levels reflect tumour burden in newly diagnosed FL patients.

**TABLE 2:** Correlation between clinical indicators and the ctDNA levels.

Clinical parameters	ctDNA mean VAF		*p*-Value
	Number (n = 28)	mean ± SEM	
**Gender**			
Male	18	13.5	0.384
Female	10	16.3	
**Age**			
<60	20	15.30	0.412
≥60	8	12.50	
**Ann Arbor stage**			
I-II	8	7.13	**0.002**
III-IV	20	17.45	
**Pathology Classification**			
1–2 grade	19	11.53	**0.019**
3 grade	9	8.75	
**FLIPI-1 Score**			
0–2	17	11.47	**0.015**
3–5	11	19.18	
**LDH (0–248 U/L)**			
Elevated	7	22.57	**0.003**
Normal	21	11.81	
**Β2M (1–2.3 mg/L)**			
Elevated	16	16.5	0.134
Normal	12	11.83	
**Bone marrow involvement**			
Positive	12	19.08	**0.010**
Negative	16	11.06	
**No. of involved areas ≥5**			
Positive	19	17.21	**0.011**
Negative	9	8.78	
**Interim assessment**			
CR	18	12.89	0.161
no-CR	10	17.40	

### 3.4 Correlation between gene mutation status and patient survival

**FIGURE 3:**
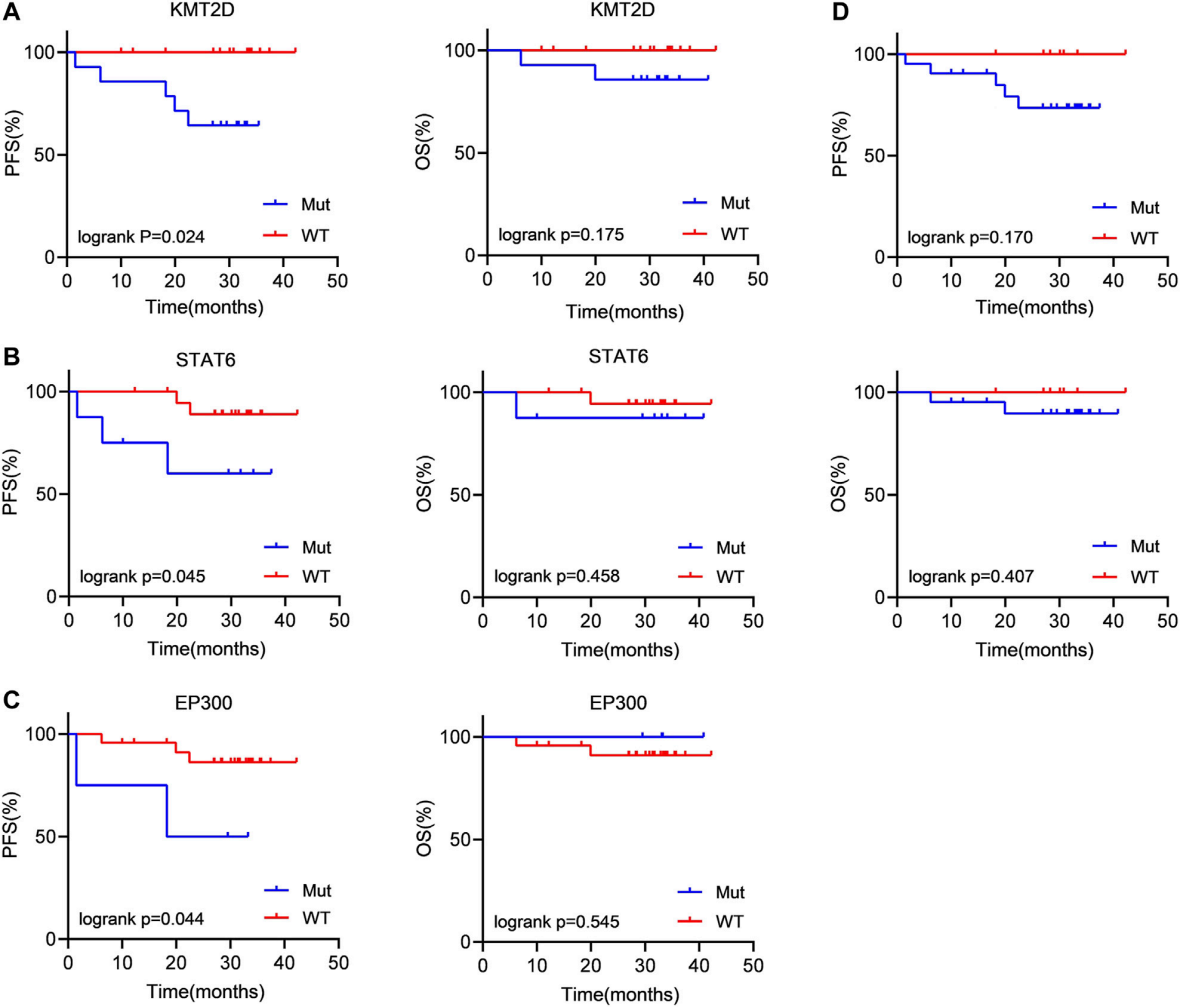
Patients carrying KMT2D, EP300 and STAT6 mutations tended to have a poor prognosis **(A–C)** Kaplan‒Meier curves were applied to compare the OS and PFS between patients carrying KMT2D, EP300 and STAT6 mutations and those without KMT2D, EP300 and STAT6 mutations. **(D)** Kaplan‒Meier curves were applied to compare the OS and PFS between patients with ctDNA mutation and without ctDNA mutation.

Then, we evaluated the impact of gene mutation status on OS and PFS in newly diagnosed FL patients using Kaplan‒Meier curves. We conducted an analysis of the survival of patients with mutated genes occurring in at least 4 cases (CREBBP, KMT2D, CARD11, STAT6, PCLO, and EP300). The results showed that KMT2D (*p* = 0.024) and STAT6 (*p* = 0.045) mutations were linked to significantly shorter PFS (Figures 3A, B). Similarly, in comparison with the patients without EP300 mutation (*p* = 0.044), the PFS was significantly shorter in the patients carrying a STAT6 mutation (Figure 3C). However, the difference in OS did not reach statistical significance. These results demonstrated that KMT2D, EP300 and STAT6 mutations predicted poor prognosis in patients with newly diagnosed FL. In addition, patients with detectable ctDNA mutations also showed a weak trend of shorter PFS than patients without ctDNA mutations. (*p* = 0.170) (Figure 3D).

## 4 Discussion

This study independently reported the prognostic value of ctDNA and the use of ctDNA to evaluate tumour burden in newly diagnosed FL patients. In detail, we analysed the characteristics of genetic mutation profiles and the clinical value of ctDNA from plasma samples in 28 newly diagnosed FL patients. Our results confirmed the relevance of high ctDNA levels and ctDNA mutation status during pretreatment in regards to Ann Arbor stage, FLIPI-1, LDH level, bone marrow involvement, pathology classification and lymph nodal involvement. In addition, we found that KMT2D, EP300 and STAT6 mutations indicated a poor prognosis in patients with FL.

We applied targeted NGS of 59 lymphoma-related genes and analysed the plasma ctDNA mutation characteristics and found that CREBBP (54%, 15/28) is the most commonly mutated gene in newly diagnosed FL patients. In fact, that was also observed in a study of the tumour tissue mutation landscape of follicular lymphoma patients (Shin et al., 2019). Moreover, the report of the tumour tissue mutation landscape in follicular lymphoma patients maintained high consistency with ctDNA mutation profiles in our study (Bastos-Oreiro et al., 2021). These results suggest that the mutation spectrum detected in plasma ctDNA samples of FL patients can reliably reflect the gene mutation information obtained from tumour tissue biopsy. However, there are also some mutated genes detected in ctDNA that are not found in tumour biopsy, which may be due to the heterogeneity of FL disease and the spatial limitations of tumour biopsy sampling. Unfortunately, neither solid nor liquid tumour biopsies can capture the complete genetic profile of the tumour, but ctDNA can be used as a supplement to bring additional mutational information because ctDNA overcomes tumour heterogeneity and contains molecular information that might be missed by examining a traditional single tumour sample.

CtDNA VAF, which is defined as the number of mutated molecules over the total number of molecules at a specific location in the genome, is closely associated with clinical features and prognosis and is considered a new biomarker of tumour burden (Arisi et al., 2022; van Velzen et al., 2022). In the present study, we found that the mean VAF was significantly increased in patients with advanced Ann Arbor stage, high-risk FLIPI, elevated serum LDH levels, bone marrow involvement, higher pathological grade and severe lymph nodal involvement. Our results suggest that ctDNA levels reflect overall tumour burden.

Moreover, patients with advanced Ann Arbor stage, high-risk FLIPI and severe lymph nodal involvement are more prone to ctDNA mutations. An unexpected finding is that ctDNA mutation generally tends to occur in FL patients with pathological grades of 1–2 rather than 3. Thus, we considered the following two aspects: 1). The 59-gene panel may not be sufficient to cover all mutated genes of FL; 2). In a recent study, gene expression profiling pointed out significant differences separating grade 1 to 2 cases from both grade 3A and grade 3B cases (Horn et al., 2018). Laurent, C et al. suggested that the high-grade FL cohort showed less frequent CREBBP mutations than grade 1–2 cases (Laurent et al., 2021). This further demonstrates the value of plasma genotyping for capturing clinically relevant tumour heterogeneity.

Dynamic ctDNA monitoring has been associated with PFS in DLBCL patients, but it is still under investigation in FL. Here, we analysed the association between ctDNA mutation status and prognosis in newly diagnosed FL patients. We found that FL patients with detectable ctDNA mutations tended to have significantly shorter PFS than those without detectable ctDNA mutations, but the difference was not statistically significant (*p* = 0.17), and we considered that the sample sizes in our study were relatively small, resulting in limited statistical power. According to previous studies, KMT2D is the most commonly mutated gene in patients with newly diagnosed FL (Morin et al., 2011), and it was also found that KMT2D mutation acts to “accelerate the mutation” and promote invasive tumour cell subpopulation formation during FL development (Green et al., 2013). Moreover, our results showed that patients with KMT2D mutations had shorter progression-free survival (PFS). Inactivating mutation of EP300 frequently occurs in DLBCL and FL, the two most common B-cell lymphomas (Pasqualucci et al., 2011). EP300 mutation has been reported to be closely related to inferior PFS (Pastore et al., 2015), which is consistent with our analysis. In addition, patients carrying STAT6 mutations had significantly shorter PFS than those without STAT6 mutations. These results suggest that KMT2D, EP300 and STAT6 mutations have a strong predictive value in the prognosis of pretreatment FL patients.

In fact, ctDNA mutations and their possible clinical applications have been initially explored in FL. Nagy, A et al. applied ddPCR to analyse the mutation and prognostic value of EZH2 in plasma samples from 4 patients with FL (Nagy et al., 2020), and Shin, S et al. discovered CREBBP mutations in plasma cfDNA using targeted deep sequencing in two follicular lymphoma patients. However, most current studies are limited to analysing single-gene mutations using liquid biopsy in FL, or other mutation signatures of ctDNA have only been characterized in a very small cohort. An important limitation of our study was the small number of cases that we were able to collect, but the clinical relevance of ctDNA was well displayed in our independent study.

## 5 Conclusion

In conclusion, our data demonstrated the feasibility of ctDNA targeted-NGS in newly diagnosed FL patients and confirmed that ctDNA mutation status and mean VAF can reflect tumour burden and are related to shorter PFS. Given the clinical relevance of the reported results, further development and validation of our findings in large patient cohorts will be needed.

## Data Availability

The data presented in the study are deposited in the National Genomics Data Center repository (https://ngdc.cncb.ac.cn/gsa-human/s/e5a7JHq7), and accession number is PRJCA015152.
