# Erratum: Application of circulating tumour DNA in terms of prognosis prediction in chinese follicular lymphoma patients

**DOI:** 10.3389/fgene.2023.1228830

**Published:** 2023-06-02

**Authors:** Frontiers Production Office

**Affiliations:** Frontiers Media SA, Lausanne, Switzerland

**Keywords:** follicular lymphoma, circulating tumour DNA, targeted next-generation sequencing, mutation, prognosis

Due to a production error, there was a mistake in Figure 1 as published. The figure was inadvertently duplicated from Supplementary Figure S1. The correct Figure 1 appears below.

**FIGURE 1:**
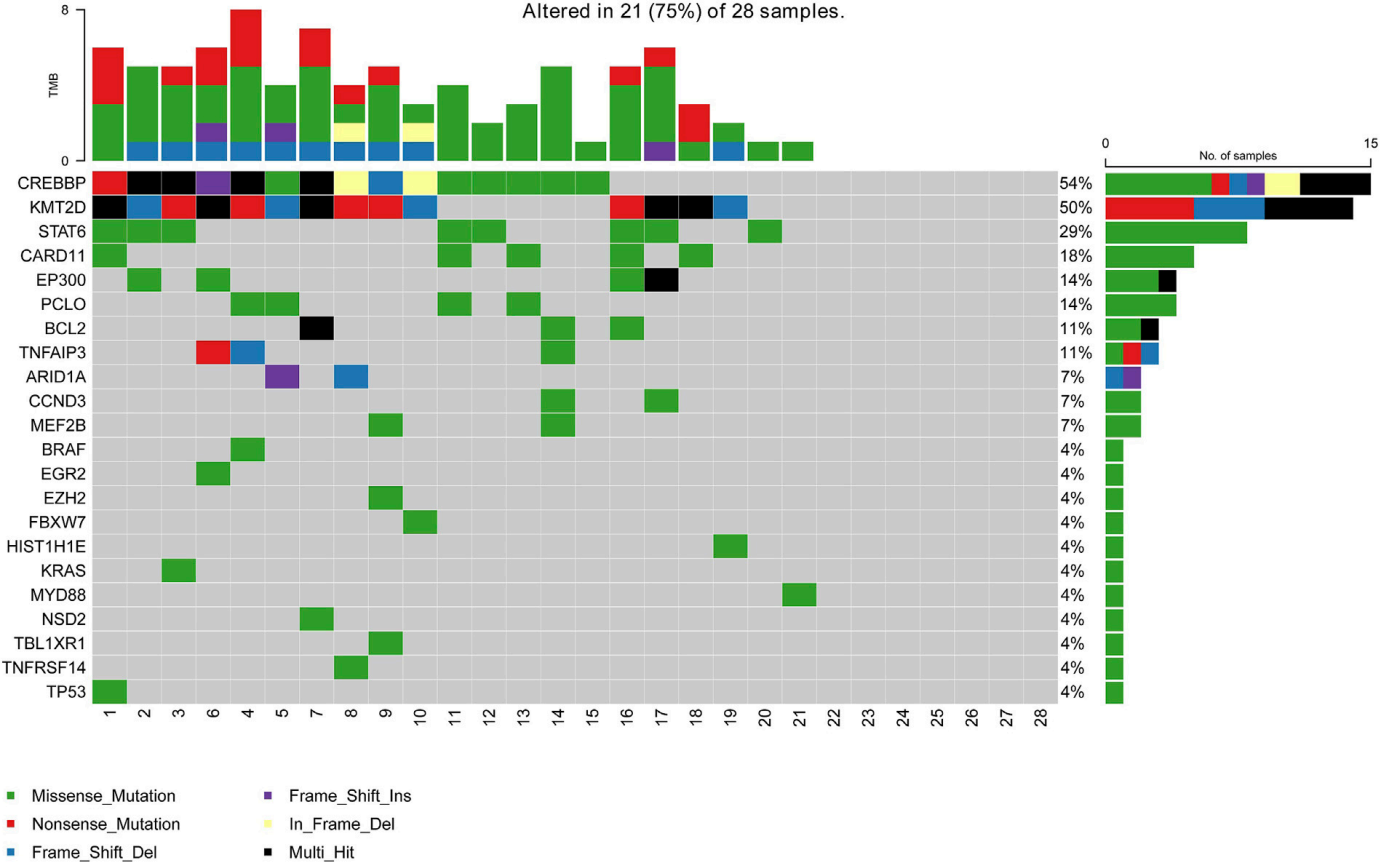
Mutation landscape of the ctDNA samples in newly diagnosed FL patients. The X-axis is the sample of individual patients. The upper histogram represents the number of mutated genes per sample. The Y-axis on the left shows the mutated genes, and the percentages on the right chart represent the mutation frequency of mutated genes.

The publisher apologizes for these mistakes. The original version of this article has been updated.

## Publisher’s note

All claims expressed in this article are solely those of the authors and do not necessarily represent those of their affiliated organizations, or those of the publisher, the editors and the reviewers. Any product that may be evaluated in this article, or claim that may be made by its manufacturer, is not guaranteed or endorsed by the publisher.

