# Supplementary material for: Application of circulating tumour DNA in terms of prognosis prediction in Chinese follicular lymphoma patients
Source: Front Genet. 2023 Apr 20;14:1066808. doi: 10.3389/fgene.2023.1066808 (PMC10157236; doi:10.3389/fgene.2023.1066808)
Supplement: Supplementary file 2 [file Table2.DOCX]

| **No.** | **Detection gene** | **No.** | **Detection gene** | **No.** | **Detection gene** | **No.** | **Detection gene** | **No.** | **Detection gene** |
| --- | --- | --- | --- | --- | --- | --- | --- | --- | --- |
| 1 | ARID1A | 13 | CD58 | 25 | IKBKB | 37 | NOTCH2 | 49 | SPEN |
| 2 | ATM | 14 | CD79B | 26 | ITPKB | 38 | NRAS | 50 | STAT6 |
| 3 | B2M | 15 | CHD2 | 27 | KLHL6 | 39 | PCLO | 51 | TBL1XR1 |
| 4 | BCL2 | 16 | CREBBP | 28 | KMT2D | 40 | PIK3CA | 52 | TCF3 |
| 5 | BCOR | 17 | DDX3X | 29 | KRAS | 41 | PIM1 | 53 | TGM7 |
| 6 | BIRC3 | 18 | EGR2 | 30 | LRP1B | 42 | POT1 | 54 | TNFAIP3 |
| 7 | BRAF | 19 | EP300 | 31 | MAP2K1 | 43 | PRDM1 | 55 | TNFRSF14 |
| 8 | CARD11 | 20 | EZH2 | 32 | MED12 | 44 | RET | 56 | TP53 |
| 9 | CCND1 | 21 | FBXW7 | 33 | MEF2B | 45 | RIPK1 | 57 | WHSC1 |
| 10 | CCND2 | 22 | GNA13 | 34 | MYC | 46 | SAMHD1 | 58 | XPO1 |
| 11 | CCND3 | 23 | HIST1H1E | 35 | MYD88 | 47 | SF3B1 | 59 | ZMYM3 |
| 12 | CD36 | 24 | ID3 | 36 | NOTCH1 | 48 | SIN3A |  |  |

**59-gene panel**
