# Supplementary figures and images for: Application of circulating tumour DNA in terms of prognosis prediction in Chinese follicular lymphoma patients
Source: Front Genet. 2023 Apr 20;14:1066808. doi: 10.3389/fgene.2023.1066808 (PMC10157236; doi:10.3389/fgene.2023.1066808)

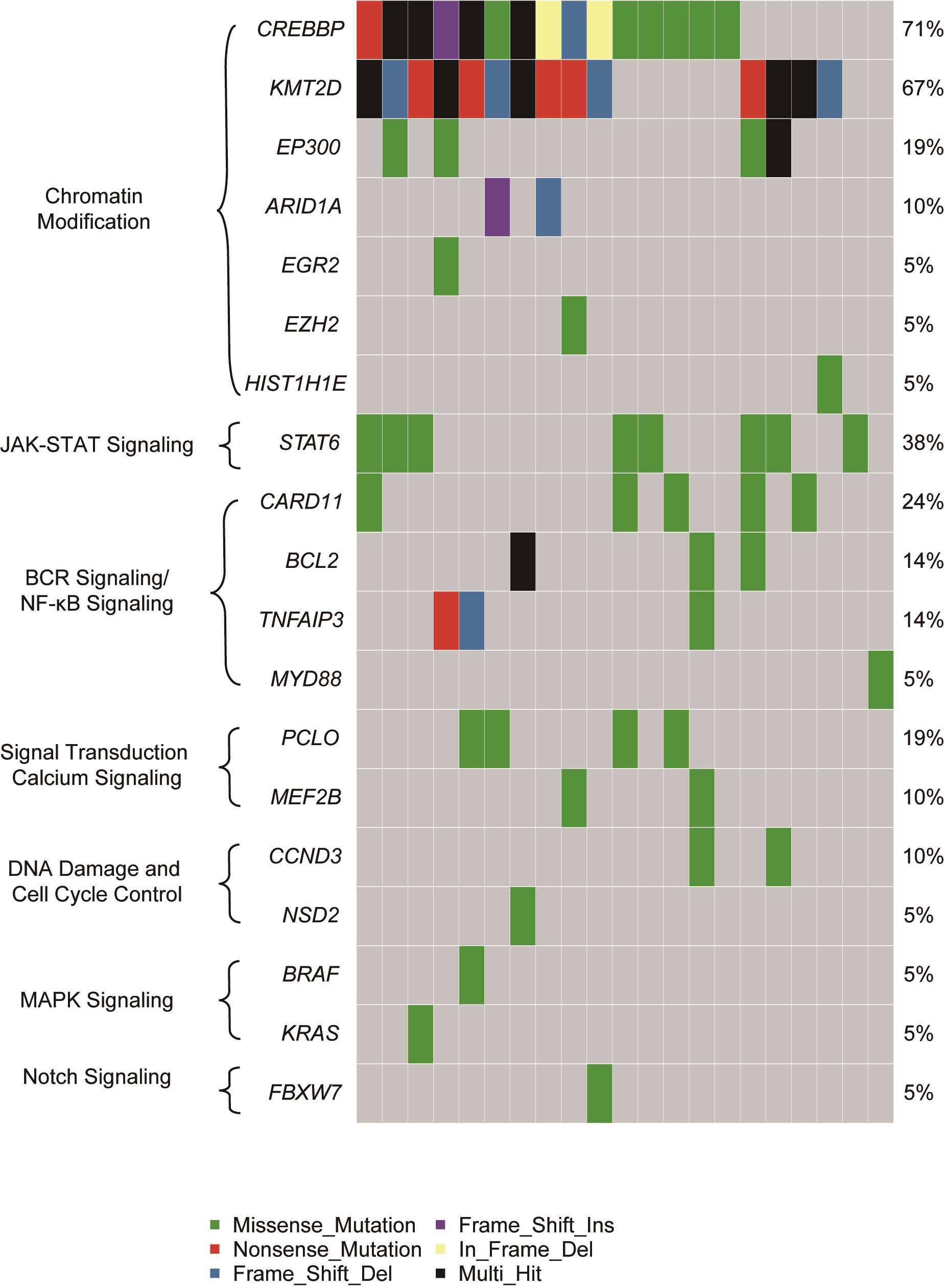

Supplement: Supplementary file 5 [file Image1.jpg]
